# Mechanical Properties of Basalt-Based Recycled Aggregate Concrete for Jeju Island

**DOI:** 10.3390/ma14185429

**Published:** 2021-09-19

**Authors:** Hong-Beom Choi, Jin-O Park, Tae-Hyun Kim, Kyeo-Re Lee

**Affiliations:** Construction Test & Assessment Center, Construction Test & Certification Department, Korea Institute of Civil Engineering and Building Technology, Goyang-si 10223, Korea; Rokmc0988@kict.re.kr (T.-H.K.); leekr@kict.re.kr (K.-R.L.)

**Keywords:** island, basalt, recycled aggregate, concrete

## Abstract

Recycled aggregate is essential to protect Jeju Island’s natural environment, but waste concrete, including porous basalt, is a factor that lowers the quality of recycled aggregate. Therefore, an experiment was conducted to analyze the properties of concrete application of basalt-based recycled aggregate (B-RA) through quality improvement. The absorption of the B-RA ranged from 3–5%; restricting its absorption to less than 3% was challenging owing to its porosity and irregular shape. However, the increase in the solid volume percentage of the concrete when replacing 25 or 50% of fresh basalt aggregate with recycled basalt aggregate improved the mechanical performance of the concrete, especially at 25%, for which a compressive strength of 55.9 MPa and modulus of elasticity of 25.9 GPa exceeded those of concrete with fresh basalt aggregate. Moreover, increasing the replacement ratio of the fresh basalt with recycled aggregate reduced the slump and decreased the air content, consequently increasing the concrete drying shrinkage. However, the replacement of fresh basalt aggregate with recycled basalt aggregate unaltered the mechanical performance of the concrete. The results indicate that efficient use of recycled aggregates can yield superior performance to that of fresh basalt, irrespective of aggregate quality.

## 1. Introduction

In South Korea, the extensive construction of concrete buildings with rapid industrialization since the 1970s has resulted in an indiscriminate collection of raw material aggregates, continuously raising the issue of environmental destruction. In particular, concerns of environmental destruction are strong in Jeju Island as its natural locales have been designated as World Natural Heritage sites. Amidst this situation, the collection of essential concrete aggregates for the infrastructure and construction industry is limited, though there is an urgent need to secure aggregates for the construction of new ports and airports. The use of recycled aggregate offers an approach to reducing environmental destruction due to raw material extraction, resulting in a green construction material called recycled aggregate concrete [1,2]. Various studies have been conducted on recycling for utilizing construction waste up to 1.3 billion tons/year globally as a valid resource [3,4,5,6,7]. Although different studies have different results, it is commonly seen that the physical properties of concrete are not significantly affected under 30% of replacement of recycled aggregates [8,9]. It is also reported that the quality of recycled aggregates affects that of concrete, and the mix design and materials of parent concrete have a significant effect [10]. The use of materials such as fiber-reinforced polymers to structurally improve concrete due to the use of relatively low-quality recycled aggregates is also being studied [11]. In addition, various studies are being conducted, including studies on the improvement of durability and fire resistance properties in preparation for disaster situations in buildings made of recycled aggregate concrete [12].

Meanwhile, the natural aggregate of Jeju Island is made of basalt, as the island was formed by long periods of volcanic activity. However, recycled aggregate from Jeju Island has not been quality-certified to be used with concrete in South Korea; thus, recycled aggregate from concrete is not used as a base course material. Although current technology is inadequate for removing cement paste and mortar from natural basalt aggregate, the basalt can qualitatively serve as the host rock for waste concrete used as recycled aggregate on Jeju Island [13,14]. According to academic literature, basalt can be categorized into 16 types of petrographic classification depending upon its quality [15], which varies significantly according environment and region because of different pore distributions. Studies have reported that basalt aggregates from Jeju Island are more porous than aggregates from other geographic regions, and that their absorption varies widely from 0–10%, whereas basalt aggregates from other countries have a low absorption (below 2%) [16,17,18,19,20,21,22]. Indeed, basalt collected from aggregate quarries on Jeju Island have been found to have an average absorption of about 3%, which is higher than that of other rock aggregates obtained in South Korea [23]. Moreover, the extensive pores present in the basalt increase its specific surface area, adversely impacting the removal of mortar from waste concrete containing basalt aggregate, which is suspected to degrade the quality of the recycled aggregate and the resulting concrete.

Several studies have been conducted on concrete using basalt as a coarse aggregate in high-strength concrete and to enhance other concrete properties based on rock classification; however, research on basalt-based recycled aggregate (B-RA) concrete is insufficient [24,25,26]. Although a previous study has analyzed the mechanical properties of concrete based on the moisture and curing conditions of B-RA found on Jeju Island, only a single type of recycled aggregate was investigated [27]. Basalt in Jeju Island requires a different approach because it has a higher pore rate than that of basalt overseas, and research is also needed to utilize irregular quality resources and recycled resources including these resources. Unlike overseas regions, basalt in Jeju Island has a more porous property, so it is believed that it may be vulnerable to elastic modulus. However, these properties may not be a big problem in Jeju Island, which uses general strength concrete because high-rise buildings were not built. In addition, it may show different properties from the decrease in the elastic modulus seen in concrete using conventional recycled aggregates. Moreover, plans to diversify the replacement ratio and quality of recycled aggregates and promote practicality must be made to accompany nature protection and infrastructure construction. Since the crushing method of B-RA with porosity affects not only the quality of the circulating aggregate but also micro cracks, a suitable method of manufacturing recycled aggregate is needed depending on properties of parent aggregate and concrete [2,28]. This study aims to evaluate the utilization of various quality B-RA into concrete aggregates and examine the effect of B-RA quality depending on method of manufacturing, replacement rate, and water-cement ratio changes on concrete quality for road pavement and secondary product concrete. Furthermore, the fresh and hardened properties of concrete specimens containing these materials were analyzed to provide basic data for a future utilization plan based on the status and quality of recycled resources. Through this series of experiments and analysis, it will be possible to confirm the availability of B-RA, replace and protect natural resources. In addition, the creation of added value through recycling of construction waste could contribute to the revitalization of related industries.

## 2. Materials and Methods

### 2.1. Experimental Plan

**Figure 1:**
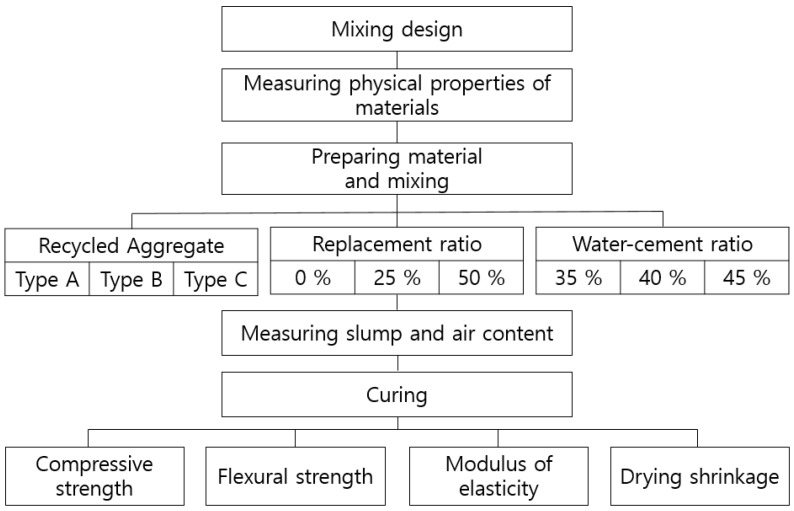
Flow chart for concrete experiment.

The objective of this study was to demonstrate and thus promote the practical use of B-RA on Jeju Island. As a basic experiment for this, it aims to apply concrete secondary products, and is an experiment to analyze the effect of the quality of recycled aggregates on concrete. The experimental plan designed for this purpose is presented in Table 1, in which inland quartz natural aggregate (NA) and basalt from Jeju Island (BA) were evaluated as base aggregates. The use of three types with different quality improving process of recycled aggregates (RA-A, -B, and -C) was evaluated as a BA replacement in ratios of 0, 25, and 50%. The quality of the recycled aggregates was enhanced using direct and indirect impact force methods. The mix proportions of the concretes containing recycled aggregate and basalt are listed in Table 2. These mixes were used to evaluate three variables: water–binder ratio, recycled aggregate type, and recycled aggregate replacement ratio. Therefore, nine mix proportions were obtained based on the Taguchi experimental design method. Its method allows to find the optimal conditions of experimental factors and levels in mix design of concrete. The three mix proportions were added using the NA. The unit cement content and sand–aggregate ratio was fixed at 350 kg/m^3^ and 45%, respectively. In experimental terms, the slump and air content were measured as the fresh concrete properties, whereas the unit mass, compressive strength, flexural strength, modulus of elasticity, and drying shrinkage as the hardened properties. Figure 1 shows flowchart for concrete experiment.

### 2.2. Materials’ Preparation

**Figure 2:**
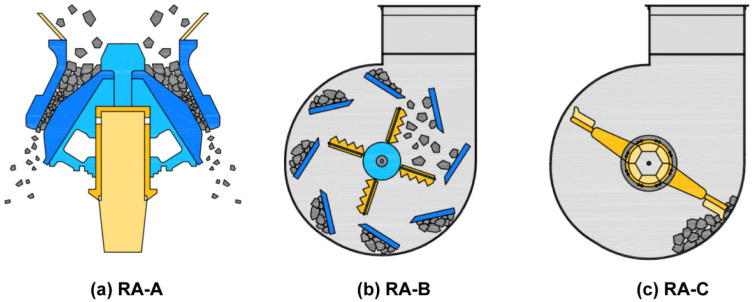
Manufacturing process of the recycled aggregate.

**Figure 3:**
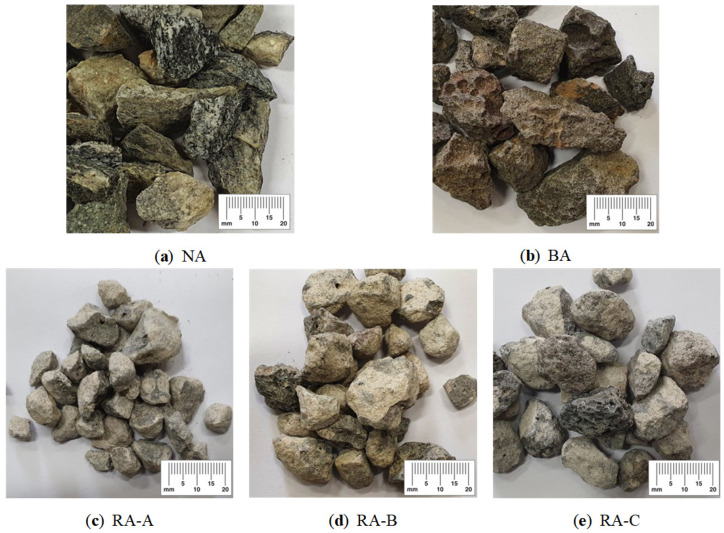
Aggregates as a function of shape and dimensions.

Type 1 ordinary Portland cement from Company S (Danyang-si, Korea) was used in the experiment, which satisfied the physical properties and chemical composition according to ASTM C150:2020 [29], as shown in Table 3 and Table 4. The compressive strengths of the cement used were 23, 29.3, and 42.5 MPa, respectively, for 3, 7, and 28 days. The initial setting was 270 min. Aggregate sizes of 25 mm were used for both the NA and BA, satisfying the requirements of ASTM C33:2018 [30]. The quartz NA was obtained from aggregate quarries in Cheonan-si. The BA was mixed from three Jeju Island quarries and exhibited an irregular shape with a porous surface and a higher absorption (2.66%) than the NA. The B-RA was made using waste concrete of Jeju Island and manufactured by a waste disposal company in Incheon Metropolitan City and Hongseong-gun. The quality of all B-RA was enhanced through processing for use in a subbase course with an absorption of 7.25%. Figure 2 shows the manufacturing process of each type of B-RA. RA-A was manufactured by crushing aggregates with direct impact force using a crusher. The RA-B manufacturing process first sends aggregates via a bucket to upper part of equipment and aggregate is free-fall to blade. After that, it produces aggregates by applying an impact force to the surface of aggregates using high-speed rotating blades and detaching plates. RA-C is prepared by crushing or scratching aggregate surfaces using the space between rotating blades and drums. The above process has been employed in manufacturing B-RA (RA-A, RA-B, RA-C) of 20 mm size. The quality of RA-A was slightly enhanced by direct impact force on the aggregate itself. RA-A of the average aggregate size was smaller than those of RA-B and RA-C. The quality of RA-B and RA-C was more enhanced by the application of indirect impact force to the surfaces of the aggregate layers. The shapes of the coarse aggregates used in the experiment are illustrated in Figure 3, and their physical properties are listed in Table 5. Although the quality-enhanced recycled aggregates exhibited varied density and absorption, their absorption failed to satisfy the quality standard for recycled aggregates regulated in South Korea (3%). Nevertheless, the quality was remarkably enhanced in compared to the raw recycled aggregate material (7.25% absorption). The impurity content of the recycled aggregate was managed to be less than 1% and used.

### 2.3. Testing Methodology

We conducted experiments in accordance with ASTM standards to analyze the fresh properties and hardened properties of the concrete. Accordingly, the slump of the fresh concrete were measured using slump cone with base diameter 200 mm, top diameter 100 mm, height 300 mm twice according to the ASTM C143:2020 [31] specifications. The air content of fresh concrete were measured using Type-B air meter with capacity of 7.0 L according to the ASTM C231:2017 [32] specifications. The concrete was maintained in a sealed state after the initial measurement. After that, the variation over time was measured at 30 min intervals up to 90 min. The compressive strengths of 100 mm diameter and height 200 mm cylindrical concrete specimens were then determined with stress rate of 0.25 MPa/s for three or more specimens at 3, 7, and 28 d of age. It was measured in accordance with ASTM C39:2020 [33] concrete compressive strength test method. The flexural strengths of 100 mm × 100 mm × 400 mm cuboid concrete specimens were determined with loading rate of 1.0 MPa/min for three or more specimens at 28 d of age. It was measured in accordance with ASTM C78:2018 [34] concrete flexural strength test method. The modulus of elasticity was measured with loading rate of 0.25 MPa/s for three or more specimens by attaching strain gauges to the 28-day-old cylindrical specimens. It was measured applying a load in accordance with ASTM C469:2014 [35] (concrete cylindrical specimen; static modulus of elasticity) and the Poisson’s ratio test method. The drying shrinkage was measured for three specimens by attaching the strain gauges that PL-60-11 of “T” company to the surface of the 100 × 100 × 400 mm cuboid specimens after demolding at 7 d of curing in accordance with ASTM C596:2018 [36] concrete drying shrinkage test method. In both strain-gauge applications, a data logger that is data logger TDS-540 of “T” company was used to acquire data under constant temperature and humidity conditions of 20 ± 2 °C and 60 ± 5%, respectively.

## 3. Results and Discussion

### 3.1. Slump

**Figure 4:**
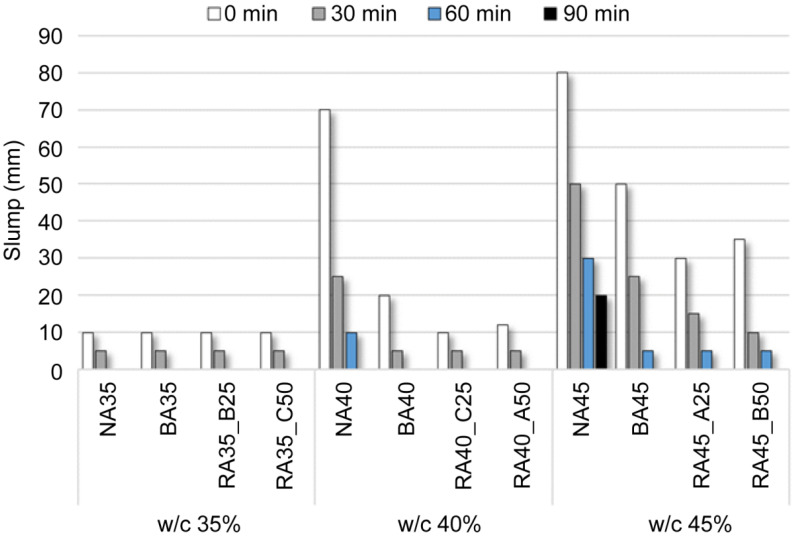
Slump of fresh concrete according to water–cement (w/c) ratio.

The slump of the concrete according to the replacement ratio and quality of the basaltic recycled aggregate is charted in Figure 4, which depicts an evident reduction in slump with decreasing water–cement ratio. Similar to existing recycled aggregate concretes, the BA concrete exhibited a decrease in slump with increasing replacement ratio of BA with B-RA [37]. These results demonstrated significant differences, including large reductions in slump over time, even for concrete samples of 100% NA and BA because the increase in aggregate specific surface area owing to the irregular shape of the BA reduced the flow-paste content. In addition, the decrease in slump could have been influenced by the high absorption of the BA and B-RA used in the experiment, as the repeated absorption and discharge of the mixed water during agitation apparently decreased the mixed water content, influencing the slump.

### 3.2. Air Content

**Figure 5:**
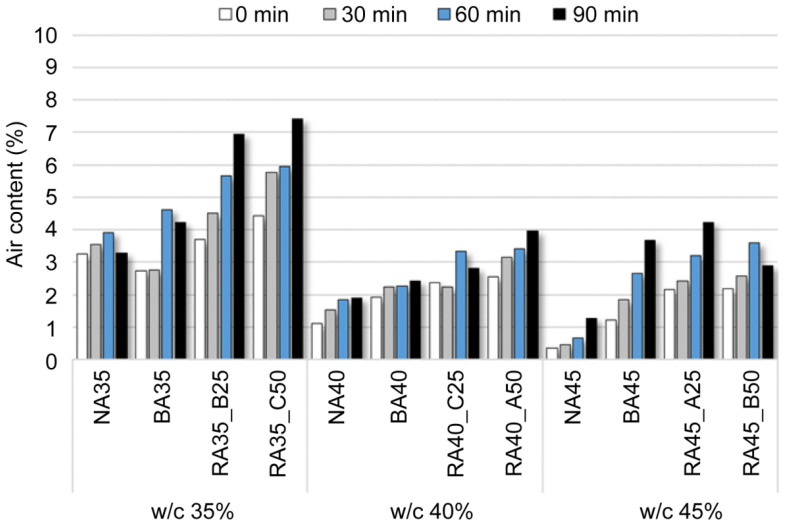
Air content of fresh concrete according to water–cement (w/c) ratio.

The air content of the fresh concrete according to B-RA quality and replacement ratio is charted in Figure 5, which indicates that the air content tended to decrease with increasing water–cement ratio owing to the improved concrete-filling ability facilitated by the corresponding increase in fluidity. In addition, the air content increased with the use of BA and larger replacement ratios of BA with B-RA. Furthermore, the rate of increase in air content over time increased for all evaluated mix proportions. This could be attributed to the absorption of mixed water by the BA and B-RA, which caused the air content to gradually increase as the moisture continuously flowed in and out of the aggregate over time [38].

### 3.3. Compressive Strength

**Figure 6:**
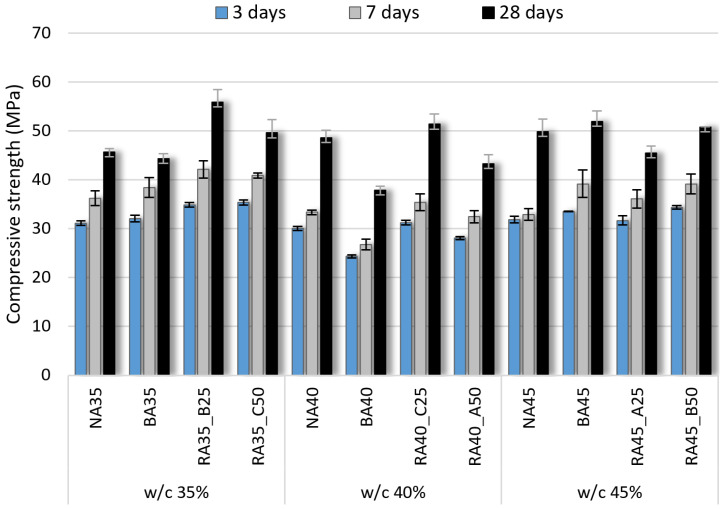
Compressive strength of concrete specimens according to water–cement (w/c) ratio.

The compressive strengths of the hardened concrete specimens according to B-RA quality and replacement ratio are charted in Figure 6. Although a reduction in the water–cement ratio could be expected to increase the compressive strength, it failed to do so because a reduction in the unit water content resulted in a degradation of the cement flow paste and deteriorated the concrete-filling ability for the same unit cement content. Therefore, the compressive strength was not significantly affected by the various mix proportions when using either NA or BA. Notably, the use of B-RA improved the concrete strength depending on its quality, because the BA exhibited a lower aggregate strength than the NA owing to the difference in porosity. This can be observed in the case of the 28-d specimens, which did not show a significant effect of strength reduction when using recycled aggregate in concrete with a compressive strength of 40~50 MPa. Moreover, the irregular shape of the BA improved the compressive strength of the concrete through increased adhesion to the paste, and the addition of B-RA with particle sizes smaller than that of the BA increased the strength by improving the aggregate performance. However, the strength was degraded when using RA-A, which had a lower quality than RA-B and RA-C. Among the recycled aggregates, the low-absorption, high-density RA-B specimens exhibited the highest compressive strength at 55.9 MPa, and the RA-C specimens displayed a higher compressive strength than the BA specimens. Therefore, replacement ratios of ≤50% can enhance the strength and quality of BA concrete containing B-RA to a certain extent.

### 3.4. Flexural Strength

**Figure 7:**
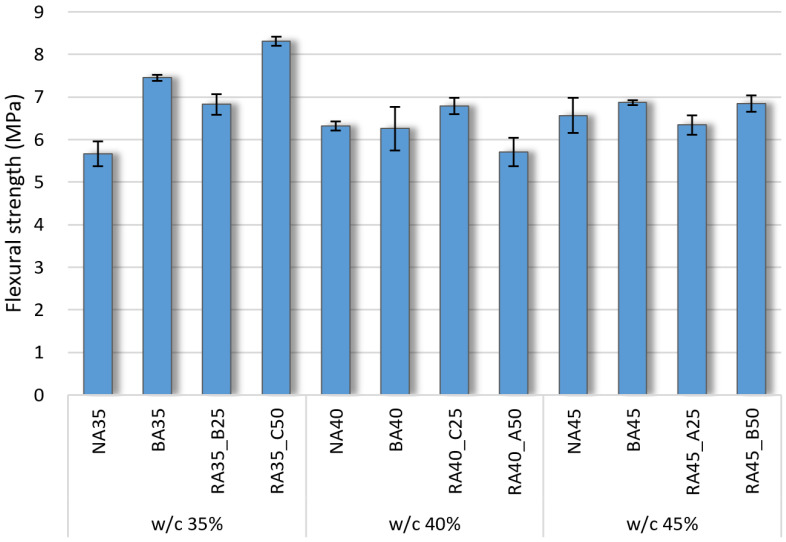
Flexural strength of concrete specimens according to water–cement (w/c) ratio.

The flexural strengths of the hardened concrete specimens according to B-RA quality and replacement ratio are depicted in Figure 7, which indicates that—similar to the compressive strength—the flexural strength was not significantly impacted by the variations in water–cement ratio, as the decreasing unit water content degraded the quality of the paste. The flexural strength of the BA specimen was higher than that of the NA specimen owing to the advantages of the irregular and elongated shape of the BA aggregate. Among the B-RA specimens, the largest flexural strength was exhibited by RA35-C50 at 8.3 MPa because of its mixture of large particle-sized aggregates; however, as shown in Table 5, the quality of the RA-C aggregate in terms of fineness modulus was 6.71, while that of RA-B aggregate was 6.91. Furthermore, the old paste on the surface of the B-RA seemingly enhanced the adhesion between the new paste and the recycled aggregate, thereby contributing to the increase in flexural strength [39,40]. Moreover, the results for the RA-A specimens indicated their vulnerability to bending stress owing to the low quality and small size of this aggregate.

### 3.5. Modulus of Elasticity

**Figure 8:**
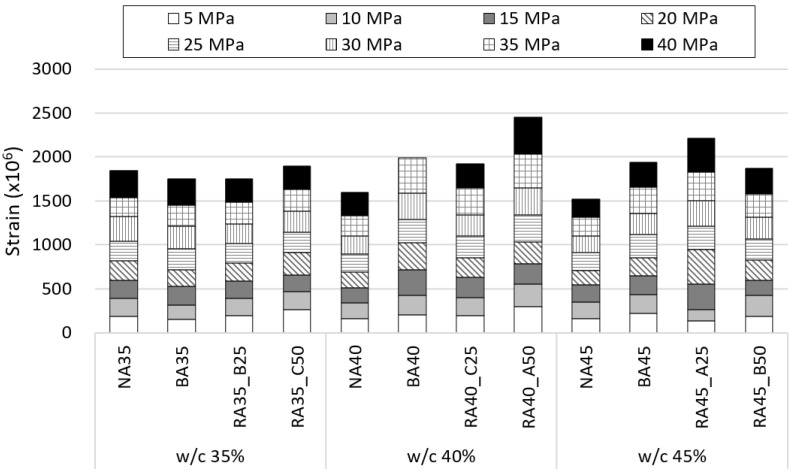
Strain of concrete specimens according to stress.

**Figure 9:**
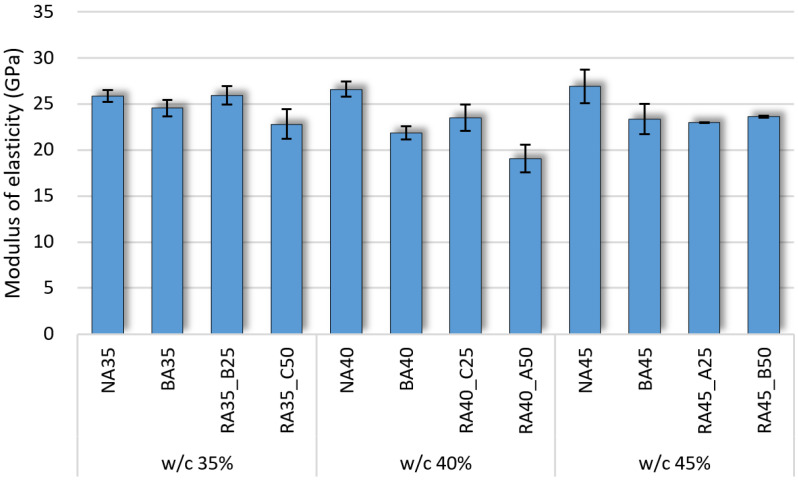
Modulus of elasticity of concrete specimens according to water–cement (w/c) ratio.

The strain of the hardened concrete specimens according to stress and the corresponding moduli of elasticity are shown in Figure 8 and Figure 9, respectively, according to B-RA quality and replacement ratio. The figures indicate that the modulus of elasticity did not vary significantly with the water–cement ratio because the reduced unit water content deteriorated the paste content and fluidity of the specimens. The modulus of elasticity of the BA specimens was lower that of the NA specimens because of the high porosity of BA, even for the same aggregate contents [41,42]. Moreover, the modulus of elasticity did not significantly decrease for a B-RA replacement ratio of 25%; instead, that of RA-B 25% actually increased to 25.9 GPa, which is almost equal to that of the NA specimen. Although the moduli of elasticity slightly decreased for the RA-B and RA-C specimens with 50% recycled aggregate, it remained in the range of 22–24 GPa. However, the modulus of elasticity of the RA-A 50% specimen significantly decreased to 19.1 GPa. Thus, the modulus of elasticity is a characteristic value that varies with the quality of the aggregate; indeed, the modulus of elasticity of porous aggregate concrete such as recycled aggregate and lightweight aggregate is known to decrease with increasing aggregate porosity [43,44,45]. Although the aggregate porosity of RA-B and RA-C was higher than that of BA, there was no considerable difference in their moduli of elasticity. Indeed, the modulus of elasticity yielded a more positive relationship in the experiment to the increasing aggregate porosity, reflecting an increase in the solid volume percentage of aggregate owing to the replacement of BA with B-RA. Accordingly, the RA-B and RA-C specimens exhibited moduli of elasticity equal to or higher than that of the BA specimens.

### 3.6. Drying Shrinkage

**Figure 10:**
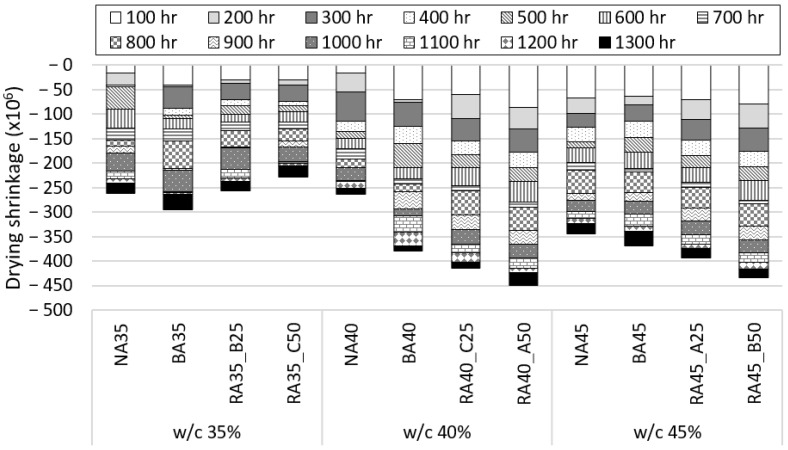
Drying shrinkage of concrete specimens according to water–cement (w/c) ratio.

The drying shrinkages of the hardened concrete specimens according to the B-RA quality and replacement ratio are charted in Figure 10, which indicates that the drying shrinkage tended to increase with increasing water–cement ratio. As drying shrinkage results from local shrinkage caused by moisture evaporation, this phenomenon is directly affected by the moisture content and is attributed to the difference in paste content. The drying shrinkage did not significantly vary with aggregate type for the 35% water–cement mix proportion, which had a small amount of excess water owing to hydration; however, the use of BA and the increasing B-RA replacement ratio increased the drying shrinkage in other mix proportions, similar to a related previous study [46]. This is because the drying shrinkage occurred as the moisture transferred to the paste evaporated from the highly absorptive aggregate; similar to the new paste, the old paste of the B-RA largely influenced the drying shrinkage.

### 3.7. Relationship between Aggregate Type and Concrete Mechanical Properties

**Figure 11:**
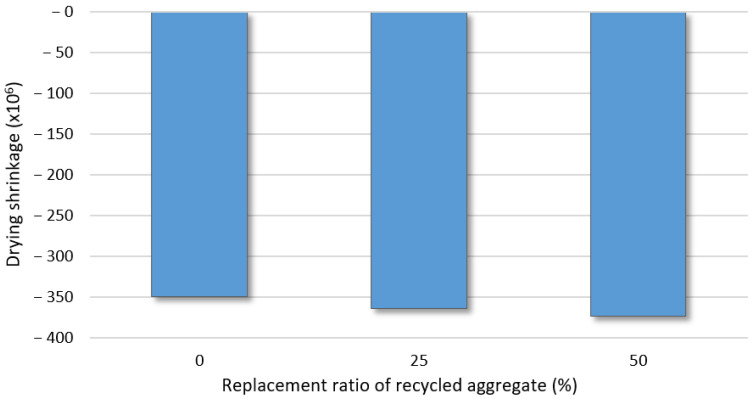
Drying shrinkage of concrete specimens according to replacement ratio of recycled aggregate.

**Figure 12:**
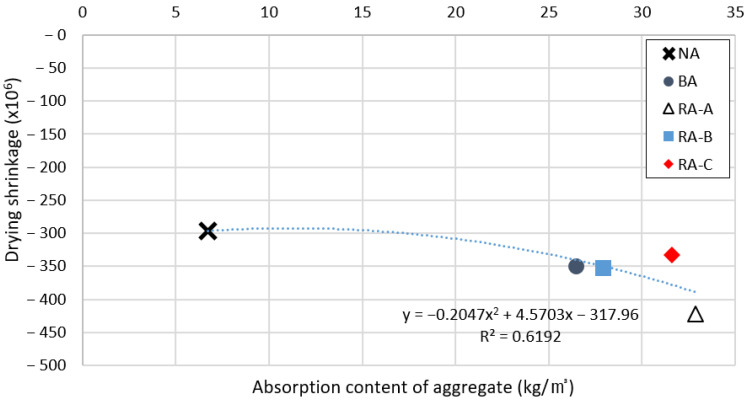
Drying shrinkage of concrete specimens according to absorption content of aggregate.

The drying shrinkage results for the concrete specimens according to the B-RA replacement ratio after 1400 h are charted in Figure 11, and the relationship between the aggregate absorption content and drying shrinkage of the concrete specimens is depicted in Figure 12. It can be observed that an increase in the B-RA replacement ratio directly increased the drying shrinkage of the concrete, because the addition of water in the process of converting the aggregates into a saturated dry-state surface increased the evaporated water content in the concrete mixing stage owing to the high absorption capacity of the recycled aggregate. Moreover, the cement paste was prone to drying shrinkage, similar to the old paste contained in the B-RA. Thus, an increase in the moisture content of the aggregate increased the drying shrinkage, exhibiting a nonlinear proportional relationship with R^2^ = 0.6192. In the figure, the absorption content of aggregate differs significant because NA aggregate exhibits a difference in absorption of aggregate of about 2% or more from those of the BA and B-RA aggregates. However, there was no significant difference between the drying shrinkages of the RA-B, RA-C, and BA specimens. As the recycled aggregates were also made from basalt, the weak paste was removed by the old-paste removal process conducted during aggregate recycling. Consequently, the relatively dense structure of the stronger old paste remaining in the RA-B and RA-C specimens did not significantly influence the drying shrinkage of the concrete.

**Figure 13:**
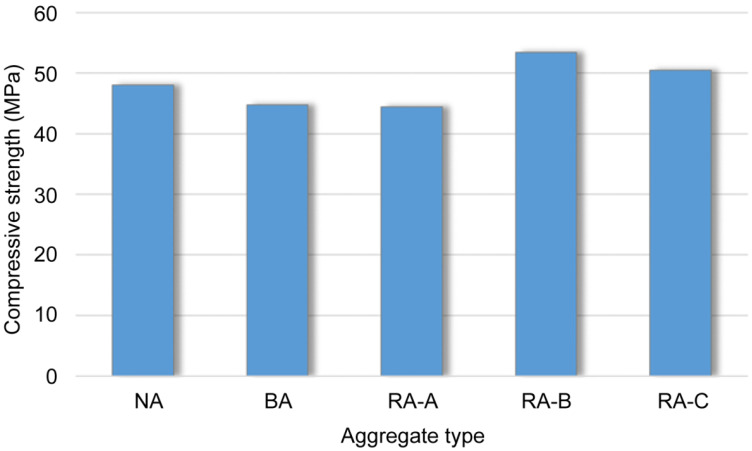
Compressive strength of concrete specimens according to aggregate type.

The average compressive strength of the concrete specimens according to the various aggregate types is shown in Figure 13, in which the NA and BA specimens do not contain recycled aggregates, whereas RA-A, RA-B and RA-C specimens contain 25–50% recycled aggregates. The BA specimen exhibited a lower compressive strength than the NA specimen and a similar strength to that of the RA-A specimen. Notably, the RA-B specimen exhibited the highest compressive strength among all specimens, though the RA-C specimen exhibited a higher compressive strength than the NA. The high porosity and low aggregate strength of the BA degraded the strength of the concrete in which it was included. However, recycled aggregates of homogeneous particle size above a certain level of quality contributed toward improving the compressive strength of the concrete by increasing the solid volume percentage of the aggregate.

**Figure 14:**
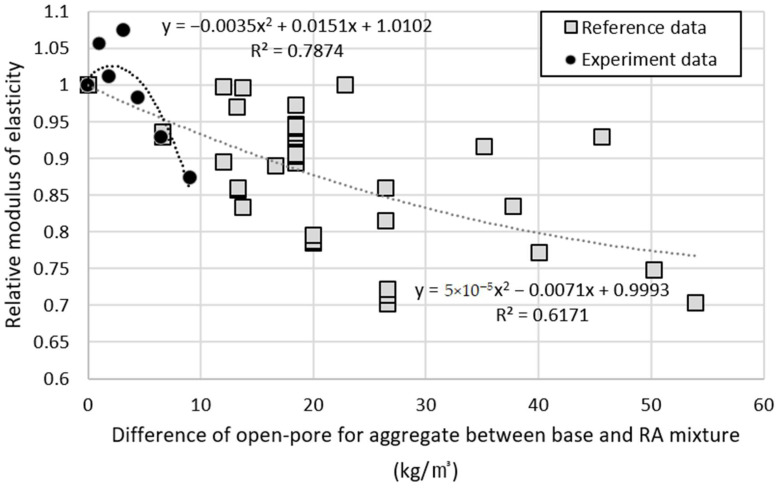
Relative elasticity modulus of basaltic recycled aggregate concrete specimens according to difference of absorption content for aggregate.

Figure 14 displays the nonlinear, inverse relation between the difference of water absorption content for aggregate and the relative modulus of elasticity of the corresponding specimens. The water absorption content of aggregate refers to the pore that the aggregate can absorb and is expressed as open-pore. The figure shows the trends of basaltic recycled aggregate concrete used in this study and those from overseas for reference [47,48,49,50,51,52,53,54,55,56,57,58,59,60,61,62,63,64]. In reference data. Even though recycled aggregates were used, the difference in absorption rates between basalt and recycled aggregates was small for specimens with high relative elastic modulus. Conversely, the specimen with a low relative elastic modulus showed a large difference in the absorption rate of aggregates, and this tendency was R^2^ value of 0.6171. The higher the similarity between the pores of natural aggregate and recycled aggregate used in mixture, the higher is the relative modulus of elasticity, and this trend was evident form the results of this experiment, with a high R^2^ value of 0.7874. The basalt used in this experiment had more pores compared to those from overseas; however, pores of the recycled aggregate used in the experiment were not much different from those of basalt. Therefore, it is believed that it has a high modulus of elasticity when 25% of the recycled aggregate is replaced.

**Figure 15:**
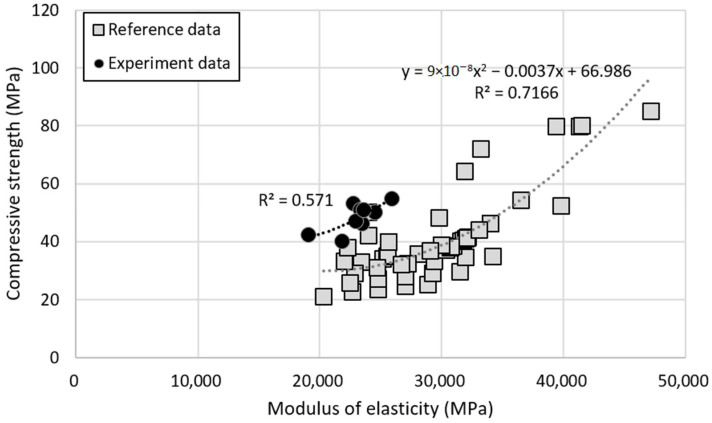
Relation between modulus of elasticity and compressive strength of recycled aggregate concrete.

Figure 15 shows the relation between compressive strength and modulus of elasticity of the specimens used in this study, compared with other reference specimens. For both experimental and reference specimens, the compressive strength and modulus of elasticity tend to be proportional, and the experimental results showed an R^2^ value of 0.571 which was somewhat lower than that from the reference results. There are several cases of improved compressive strength via replacement of recycled aggregates that do have small difference in quality from natural aggregates in the region [65,66,67]. Using concrete with high strength is expected to compensate for the reduction in modulus of elasticity exhibited in recycled aggregate concrete. Although the compressive strength of the experimental data and the reference data specimen was similar, the elastic modulus showed a large difference. This trend is due to the properties of natural basalt. The absorption of basalt in the experimental data was 2.66%, while most absorption of basalt in the reference data was low less than 1%. In areas where natural aggregates with relatively high absorption rates exist, the increase in absorption due to the adhesion of old mortar is expected to have a relatively small effect on the physical properties of concrete.

## 4. Conclusions

The basic properties of concretes mixed with porous B-RA of various qualities, in different replacement ratios, having different water–cement ratios were analyzed to investigate the effects of its use in concrete. The conclusions of this study are as follows.

1. The fresh slump and air content were deteriorated because of moisture movement between B-RA and cement paste, which resulted in generation of air regardless of the quality of B-RA. This trend became more pronounced over time.
2. The compressive strengths of the concrete specimens increased with the use of low-absorption B-RA due to stability of aggregate strength and improvement of solid content; the compressive strength was the highest at 55.9 MPa for a 25% replacement ratio of BA with RA-B. Similarly, the use of recycled aggregates with a large, uniform particle size improved the flexural strength, which was not significantly influenced by the mix proportions otherwise.
3. Although the elastic modulus is known to be affected by the voids of aggregates, the difference between the pore of BA and B-RA used in this experiment is not significant; therefore, it is judged that the strength improvement due to the B-RA with low absorption results in increased modulus of elasticity. The modulus of elasticity was the highest at 25.9 GPa with 25% RA-B replacement and slightly decreased when using 50% RA-B replacement.
4. The drying shrinkage of concrete tended to decrease with decreasing water–cement ratio and slightly increased with increasing replacement ratio of BA with B-RA due to increase in pore content caused by usage of old paste, which affects drying shrinkage. However, there was no significant difference between the drying shrinkage of specimens containing 100% BA and 25% replacement with B-RA, which had an absorption rate of 3–4%. In addition, the influence of the old paste was insignificant when using recycled aggregates with the paste still attached to porous basalt.
5. Although the recycled aggregates used in these experiments did not exhibit an absorption of 3% or less, as dictated by the Korean standard for recycled aggregates, the use of quality-enhanced recycled aggregates, which slightly differ in quality compared to local natural aggregate (at replacement ratios less than 50%), can provide a greater improvement in strength and modulus of elasticity of concrete than that be obtained when using only BA. Thus, the fresh properties and drying shrinkage must still be improved to the level exhibited by general recycled aggregate concrete before natural BA can be utilized as an effective source of replacement aggregate. As such, future studies should focus on enhancing aggregate quality, developing various improved curing methods, and evaluating effective binders for B-RA concretes.
6. Recycled aggregate in areas with low quality of natural aggregate is also believed to be used as an aggregate for concrete as it improves the quality. If the quality classification varies for various recycled aggregates as well as by-product aggregates, it is believed that aggregate shortages can be addressed by using them as various alternative aggregates, such as concrete, asphalt, block and subbase. Even with the use of alternative aggregates, it will be possible to reduce the unit price by more than 10% to manufacture concrete with the same quality as the use of natural resources. Through this result, it will be possible to use it as secondary product concrete such as interlocking blocks and water pipes. In addition, it is judged that there is a possibility of applying concrete for road pavement through additional durability improvement studies.

## Figures and Tables

**Table 1:** Experimental parameters.

Aggregate Type	Replacement of BA with Recycled Aggregate (%)	Water–Cement Ratio (%)
Natural aggregate (NA, Inland) Basalt aggregate (BA) Recycled aggregate type A (RA-A) Recycled aggregate type B (RA-B) Recycled aggregate type C (RA-C)	0 25 50	35 40 45

**Table 2:** Concrete mix proportions.

Mixture	w/c (%)	s/a (%)	Unit Weight (kg/m^3^)								
			Water	Cement	Sand	Coarse Aggregate					Total
						NA	BA	RA-A	RA-B	RA-C	
NA35	35	45	123	350	825	1072	–	–	–	–	2369
BA35			123	350	825	–	1018	–	–	–	2316
RA35-B25			123	350	825	–	764	–	251	–	2312
RA35-C50			123	350	825	–	509	–	–	484	2291
NA40	40		140	350	805	1046	–	–	–	–	2341
BA40			140	350	805	–	993	–	–	–	2288
RA40-C25			140	350	805	–	745	–	–	236	2276
RA40-A50			140	350	805	–	497	471	–	–	2263
NA45	45		150	350	785	1019	–	–	–	–	2312
BA45			150	350	785	–	969	–	–	–	2261
RA45-A25			150	350	785	–	726	230	–	–	2249
RA45-B50			150	350	785	–	484	–	477	–	2254

**Table 3:** Physical properties of cement.

Density (g/cm^3^)	Fineness (cm^2^/g)	Setting Time (h)		Compressive Strength (MPa)		
		Initial	Final	3 d	7 d	28 d
3.15	3818	4.5	7.15	23.0	29.3	42.5

**Table 4:** Chemical composition of cement.

CaO (%)	SiO_2_ (%)	Al_2_O_3_ (%)	Fe_2_O_3_ (%)	MgO (%)	Ignition Loss (%)	Misc. (%)	Total (%)
62.44	21.12	4.40	3.19	3.10	3.36	2.39	100

**Table 5:** Physical properties of coarse aggregates.

Aggregate Type	G_max_ (mm)	Oven-Dry Density (g/cm^3^)	Absorption (%)	Bulk Density (kg/m^3^)	Solid Content (%)	Fineness Modulus
NA	25	2.72	0.64	1564.2	57.5	7.34
BA	25	2.58	2.66	1396.0	54.2	6.96
RA-A	20	2.45	4.72	1431.7	57.8	6.18
RA-B	20	2.55	3.10	1522.4	59.7	6.91
RA-C	20	2.46	4.14	1517.4	61.7	6.71

G_max_ = Maximum size.

## Data Availability

Not applicable.
